# Increasing the availability and utilization of reliable data on population micronutrient (MN) status globally: the MN Data Generation Initiative

**DOI:** 10.1093/ajcn/nqab173

**Published:** 2021-05-24

**Authors:** Kenneth H Brown, Sophie E Moore, Sonja Y Hess, Christine M McDonald, Kerry S Jones, Sarah R Meadows, Mari S Manger, Jennifer Coates, Silvia Alayon, Saskia J M Osendarp

**Affiliations:** Department of Nutrition and Institute for Global Nutrition, University of California Davis, Davis, CA, USA; Department of Women's and Children's Health, Kings College London, London, United Kingdom; Medical Research Council Unit, The Gambia at the London School of Hygiene and Tropical Medicine, Fajara, The Gambia; Department of Nutrition and Institute for Global Nutrition, University of California Davis, Davis, CA, USA; Department of Pediatrics, University of California San Francisco School of Medicine, San Francisco, CA, USA; International Zinc Nutrition Consultative Group, San Francisco, CA, USA; National Institute for Health Research Biomedical Research Centre Nutritional Biomarker Laboratory, Medical Research Council Epidemiology Unit, University of Cambridge, Cambridge, United Kingdom; National Institute for Health Research Biomedical Research Centre Nutritional Biomarker Laboratory, Medical Research Council Epidemiology Unit, University of Cambridge, Cambridge, United Kingdom; International Zinc Nutrition Consultative Group, San Francisco, CA, USA; Friedman School of Nutrition Science and Policy, Tufts University, Boston, MA, USA; United States Agency for International Development Advancing Nutrition, Arlington, VA, USA; Save the Children, Washington, DC, USA; The Micronutrient Forum, Washington, DC, USA

**Keywords:** vitamin deficiency, mineral deficiency, nutrition biomarkers, nutritional status assessment, nutrition surveys, laboratory quality assurance

## Abstract

Micronutrient (MN) deficiencies can produce a broad array of adverse health and functional outcomes. Young, preschool children and women of reproductive age in low- and middle-income countries are most affected by these deficiencies, but the true magnitude of the problems and their related disease burdens remain uncertain because of the dearth of reliable biomarker information on population MN status. The reasons for this lack of information include a limited understanding by policy makers of the importance of MNs for human health and the usefulness of information on MN status for program planning and management; insufficient professional capacity to advocate for this information and design and implement related MN status surveys; high costs and logistical constraints involved in specimen collection, transport, storage, and laboratory analyses; poor access to adequately equipped and staffed laboratories to complete the analyses reliably; and inadequate capacity to interpret and apply this information for public health program design and evaluation. This report describes the current situation with regard to data availability, the reasons for the lack of relevant information, and the steps needed to correct this situation, including implementation of a multi-component MN Data Generation Initiative to advocate for critical data collection and provide related technical assistance, laboratory services, professional training, and financial support.

## Background

Deficiencies of specific vitamins and minerals are responsible for a sizeable share of the mortality and morbidity experienced by young children and women of reproductive age in low- and middle-income countries (LMICs). For example, deficiencies of vitamin A, zinc, and vitamin D each increase the susceptibility to and severity of common infections, like diarrhea and pneumonia, which are responsible for a large proportion of child deaths in LMICs (1–5). Peri-conceptional maternal folate insufficiency increases the risk of infant neural tube defects (NTDs), including anencephaly and spina bifida, resulting in both stillbirths and postnatal deaths, as well as physical disabilities of surviving infants (6, 7). Maternal iron deficiency anemia during pregnancy is associated with an increased incidence of low birth weight (8, 9), thereby contributing to infant mortality, impaired postnatal growth, and a heightened risk of metabolic diseases as adults; severe maternal anemia of all causes during pregnancy increases the risk of maternal death (10). However, the total disease burden attributable to micronutrient (MN) deficiencies remains uncertain due to the scarcity of information on population MN statuses.

According to the 2013 Lancet Nutrition Series (11), each year more than 425,000 deaths of children less than 5 years of age are due to maternal or child MN deficiencies. Another estimate, which considered a broader set of MN deficiencies and applied somewhat different assumptions, concluded that as many as 745,000 under-5 deaths may occur each year due to MN deficiencies (12). Even this latter figure may underestimate the actual mortality burden, as it did not include an updated estimate of NTD deaths due to maternal folate insufficiency (6), the increasingly recognized problem of deaths due to thiamine deficiency and infantile beriberi (13–15), or the possible effects of vitamin D deficiency on the risk of acute respiratory infections (5). In addition to the impact of MN deficiencies on child mortality, selected deficiencies adversely affect neuro-behavioral and cognitive development. For example, both iodine deficiency and iron deficiency anemia impair children's cognitive development, as well as adult cognitive function (16, 17). Other MN deficiencies, like thiamine and vitamin B12 deficiencies, affect neurological function and social and educational performance (18, 19).

There are 5 major reasons why greater availability of high-quality information on population MN statuses is critical for establishing coherent MN deficiency control programs. First, reliable, population-level information is needed to define whether a deficiency problem exists in a particular population and whether the prevalence is of sufficient magnitude to justify a public health prevention program rather than just individualized treatment of sporadic cases of deficiency. Second, data are needed on the population subgroups most affected to enable appropriate targeting of interventions to maximize program efficiency and avoid unnecessary (and costly) program outreach. Third, data are needed to gauge whether the programs are achieving their desired outcomes and to track progress against the United Nations’ Sustainable Development Goals (20). Fourth, for those MNs that produce adverse effects when consumed in excessive amounts, it is important to monitor for any possible risks of toxicity imposed by these programs. Finally, the information is useful for research purposes to determine the relationships between MN statuses and a variety of health outcomes.

There is ample evidence to indicate that the availability of data can instigate programmatic action, drive program modifications, and contribute to greater cost-effectiveness and safety of these programs. To cite just a few examples, availability of information on population median urinary iodine concentrations (UICs) and/or newborn thyrotropin levels has inspired multiple countries to initiate (or reinitiate) iodine intervention programs and has provided countries with the rationale for increasing or decreasing the level of salt iodization, as needed, to maximize program impact and ensure safety (21–24). Similarly, data on the prevalence of vitamin A deficiency in Guatemala motivated research on vitamin A fortification, which led to a national sugar fortification program and subsequent modifications of other vitamin A–deficiency control activities (25). Data on RBC folate statuses from the US National Health and Nutrition Examination Survey have allowed the CDC to identify population subgroups with suboptimal RBC folate concentrations who need to be targeted with additional interventions beyond just wheat flour fortification to prevent NTDs (26).

With respect to potential opportunities for cost savings, a recent set of analyses using vitamin A biomarker and dietary data and vitamin A program delivery costs in Cameroon found that it was possible to reimagine the existing vitamin A intervention programs and achieve the same level of effective coverage by expanding food fortification nationally and reducing the scope of vitamin A supplementation in areas with a better vitamin A status (12, 27, 28). These program modifications could save more than 16 million dollars in program costs over 10 years, whereas the data that enabled this modeling were collected and analyzed for less than 1 million dollars. Thus, investment in data could more than pay for itself through greater program efficiency. Likewise, data on improved vitamin A statuses in Guatemala following revitalization of the sugar fortification program have allowed the government to scale back vitamin A supplementation, thereby reducing the risk of vitamin A toxicity while lowering program costs (29). Similarly, findings on adequate UICs following salt iodization in Nepal allowed the country to discontinue the iodized oil capsule program (30). Thus, the availability of data on population MN statuses has not only presented opportunities for MN intervention programs and improvements of these programs, but has also led to safer and more coherent interventions and considerable cost savings overall.

Regrettably, representative national and subnational MN biomarker data are very limited, not only with regard to the number of countries that have generated relevant information but also the frequency of data collection, the number of MNs considered, and the analysis, interpretation, and utilization of the data once they become available. As a result, public health programs are not always deployed where and when they are needed, and these programs are often less cost-effective and sometimes riskier than they could be. To address this issue of data scarcity, the Micronutrient Forum assembled a Core Working Group of experts in MN deficiencies, assessments of population nutritional statuses, laboratory analyses of MN biomarkers, and related public health programs to *1*) describe existing information gaps; and *2*) propose steps that should be taken to promote and support the collection, interpretation, and dissemination of more high-quality information on population MN statuses. The current review presents the findings and recommendations of the working group, which were further informed by a broader multi-stakeholder advisory group comprised of experts in specific MNs and representatives of national governments in LMICs, bilateral and multi-lateral technical assistance agencies, and private foundations, who reviewed a preliminary draft of the proposed strategy and provided critical inputs to the final plan described herein (see **Supplemental Table 1** for a list of Advisory Group members). The full working group report can be found on the Micronutrient Forum website (31).

The planned initiative focuses on a particular subset of MNs because of their likely high prevalence of deficiency and/or the severe clinical or functional responses to a poor status. Specifically, we considered vitamin A, folate, vitamin B12, vitamin D, thiamine, iodine, iron, and zinc, whose deficiencies may result in physical disability, sensory impairments, restricted physical growth, impaired neuro-cognitive development, or death. The recommended biomarkers for these MNs, as published by several expert groups (32–49), are listed in **Table 1**, and the suggested laboratory methods for analyzing these biomarkers have been compiled on the OpeN-Global web site (50) and in the working group report. Other MNs, like riboflavin, niacin, pyridoxine, and selected mineral elements, such as selenium, may be equally important for public health but are not yet being prioritized either because of the paucity of information on population status or an incomplete understanding of the health implications of deficiency. Calcium is also considered to be a key nutrient of public health importance, both for the prevention of rickets in children and preeclampsia in pregnant women, but calcium was not addressed by the working group because there are no easily measured biomarkers of calcium status, so population assessments of the risk of deficiency are based primarily on dietary intake data.

**TABLE 1:** Key biomarkers recommended for assessing status of selected MNs by BOND, NYAS, WHO, and other expert groups

Nutrient	Primary recommended biomarkers	Expert groups	Refs
Vitamin A^1^	s/p retinol, retinol binding protein	BOND, WHO	(32, 33)
	total body stores (retinol isotope dilution method)	IAEA	(34)
Thiamine	RBC or whole blood thiamine diphosphate, RBC transketolase activity	Global Thiamine Alliance	(35)
Folate	RBC/s/p folate	BOND, WHO, Folate Task Team	(36–39)
Vitamin B12	s/p cobalamin, transcobalamin-2, methyl malonic acid	BOND, WHO	(36, 40)
Vitamin D	s/p 25-OH vitamin D	Multiple [see Roth et al. (41)]	(41)
Iodine	urinary iodine, s/p thyroglobulin	BOND, IGN, WHO	(42, 43)
Iron^1^	s/p ferritin, soluble transferrin receptor, transferrin saturation, RBC zinc protoporphyrin	BOND, WHO	(44–47)
Zinc^1^	s/p zinc	BOND, IZiNCG	(48, 49)

Abbreviations: BOND, Biomarkers of Nutrition for Development; IAEA, International Atomic Energy Agency; IGN, Iodine Global Network; IZiNCG, International Zinc Nutrition Consultative Group; MN, micronutrient; NYAS, New York Academy of Sciences; RBP, retinol-binding protein; s/p, serum or plasma can be used for analyses.

1 Markers of inflammation should also be measured to help with the interpretation of status markers for vitamin A (retinol and RBP), iron (ferritin), and zinc (serum zinc concentration).

### Current situation regarding data on MN statuses of populations

The most comprehensive, publicly accessible source of nationally representative information on MN statuses is the WHO's Vitamin and Mineral Nutrition Information System (VMNIS) (51). Although some national survey data are not included in this database, either because the WHO is unaware of a particular survey or has not yet curated and published the data or because the country has decided not to provide the information for public dissemination, the VMNIS database is reasonably complete and provides information on MN statuses in LMICs. The Iodine Global Network (IGN) provides separate, more comprehensive updates on national iodine status, so the IGN “scorecard of iodine nutrition” was also consulted for the current report (52).

**FIGURE 1:**
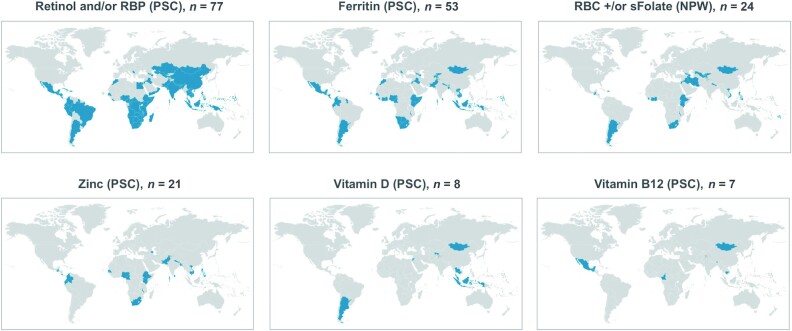
Number of low- and middle-income countries with specified data on MN status of selected population subgroups since 1980, according to the WHO's Vitamin and Mineral Nutrition Information System. Data are from the WHO (51), downloaded from the WHO website on 28 February 2020. Data from high-income countries were not included in tallies. Abbreviations: MN, micronutrient; NPW, nonpregnant women of reproductive age; PSC, preschool-age children; RBP, retinol-binding protein.

The World Bank classifies countries as upper-income if the annual gross national income (GNI) per capita is >US$12,375, and all countries with a GNI less than that figure are considered LMICs. We examined how many of the 138 LMICs have data on specific MN status biomarkers in the VMNIS database. The amount of information available in the VMNIS on specific MN deficiencies among preschool children (PSC) in LMICs varies by MN (**Figure 1**). From 1988–2018, 77 LMICs (55.8%) reported data on vitamin A statuses, using either the serum retinol or retinol binding protein concentration; 53 (38.4%) reported on iron statuses using serum ferritin; 21 (15.2%) reported on serum zinc; 8 (5.8%) reported on vitamin D; and 7 (5.1%) reported on vitamin B_12_. As information on folate statuses is more critical for women of reproductive age, we examined the respective availability of information on folate statuses in the VMNIS database. Data on RBC or serum folate among nonpregnant women of reproductive age are available for a total of just 24 LMICs (17.4%). Only a handful of LMICs have generated information on thiamine, riboflavin, or selenium statuses of PSC, but data for these nutrients are not yet reported in the VMNIS.

To assess the frequency of MN status data collection for those countries that have produced data, we focused on 2 MNs for which there is relatively more information: namely, vitamin A and iron. In 2015, Stevens et al. (3) reported that only 83 countries produced relevant information for vitamin A deficiency among PSC during the years 1990 to 2013, of which just 29 conducted more than 1 survey during this 23-year period. We also reviewed the VMNIS database and found that of the 52 countries that provided data on PSC's iron statuses from 1988 to 2018, 39 countries (75%) completed just 1 survey during this 30-year period, 12 countries completed 2 surveys, and 1 completed 3. The average year of the last survey for those countries that completed a single survey was 2005, and the average number of years between surveys was 9 years for those countries that completed 2 surveys. In summary, not only is there a paucity of information for most MN status biomarkers, but the information that is available is often outdated.

These sets of findings are consistent with the conclusions of the latest Lancet Series on progress in maternal and child undernutrition (53), which stated that national and local governments and their development partners need information from national household surveys for purposes of advocacy, strategy development, monitoring, and evaluation. The papers highlighted the data scarcity related to MN deficiencies, despite their significant impacts on health and productivity, and the authors emphasized the need for renewed efforts and funding to fill the vast data gap.

Notably, the situation regarding data on iodine statuses is different from that of other MNs. The iodine statuses of populations are generally assessed by measuring UICs among school-age children, although recently more attention has also been directed to women of reproductive age. According to the IGN, since 1994 a total of 126 LMICs (91%) have produced information on children's iodine statuses, and 113 of these countries (82% of all LMICs) have generated data within the past 15 years (52).

**FIGURE 2:**
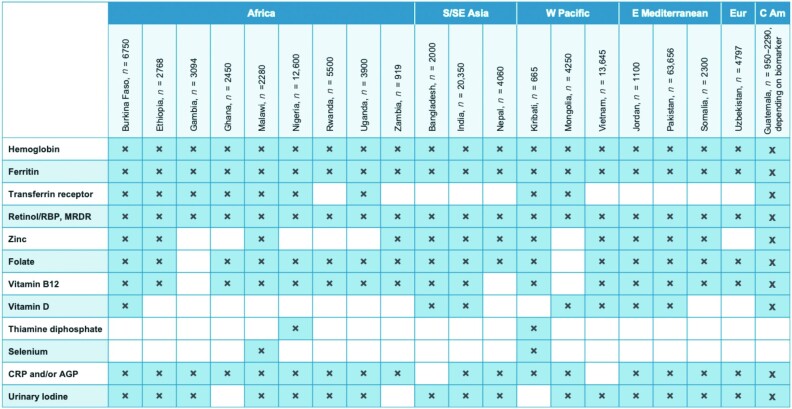
MN and inflammation biomarkers included in national surveys completed from 2015–2019 or currently being planned; *n* = total number of specimens intended for analysis for women of reproductive age and preschool children combined. (Urinary iodine was generally analyzed in school-age children.) Guatemala data are from individual rounds of a national surveillance system, reflecting the period from 2015–2019. Abbreviations: AGP, alpha-1 acid glycoprotein; CRP, C-reactive protein; MN, micronutrient; MRDR, Modified Relative Dose Response test; RBP, retinol-binding protein.

Ideally, MN status surveys should be completed every 5 years to coincide with the formulation of national nutrition and health plans. Based on reports provided by key informants from international technical assistance and donor agencies and representatives of governments and research institutions in LMICs, we compiled information on the most recently conducted or currently planned surveys in LMICs that included or will include an MN status assessment. Specifically, we focused on surveys that were completed during the past 5 years or are currently in the planning or implementation stage. Our search found 21 surveys in LMICs that met these criteria, of which 9 took place or are soon to be carried out in Africa, 3 in South or Southeast Asia, 3 in the Western Pacific, 3 in the Eastern Mediterranean, and 1 each in Europe and Central America (**Figure 2**). In other words, just 15% of LMICs generated MN status data during this period. Of the 21 surveys that were identified, we were able to obtain final or provisional lists of MN biomarkers for 20 surveys, all of which included assessments of iron and vitamin A statuses. Folate statuses were assessed or scheduled for assessment in 18 countries, vitamin B_12_ in 17 countries, iodine in 16 countries, and zinc in 13. Eight countries completed or planned assessments of vitamin D statuses, and 2 countries each assessed selenium or thiamine statuses.

The number of recent surveys identified suggests that each year only about 4 new surveys that include MN status biomarkers are being conducted in LMICs, with sample sizes ranging from 490–6840 women and a similar number of PSC (mean = ∼2000 women and ∼2000 PSC per survey), except for the most recent surveys in India and Pakistan, which each enrolled more than 20,000 participants (Figure 2). Some countries have also collected specimens from school-age children (particularly in the case of iodine assessments), adolescents, and adult males, so the total number of specimens actually collected in each country may be greater than the total number of specimens shown in Figure 2, which just reflects the analyses planned for women and PSC. The recent surveys included a broader range of MN status biomarkers than was the case previously, and they generally included biomarkers of inflammation (18 of 20 surveys), which are helpful for interpreting some of the MN status biomarkers (54–59). Based on the average sample sizes for women and PSC included in these national surveys (excluding India and Pakistan), the recent surveys generated a mean of ∼4000 sets of specimens from these 2 population subgroups combined. This number multiplied by an estimated 4 surveys completed annually indicates that the total laboratory throughput for these population subgroups each year is approximately 16,000 analyses for each biomarker assessed, or twice that number if analyzed in duplicate. This information can be used to project the additional number of samples that might be generated in the future as efforts to amplify the annual number of surveys become more successful.

By way of comparison, we also searched the number of selected other health surveys completed during the same period (2015–2019). A total of 63 nationally representative Demographic and Health Surveys (DHS) surveys or Malaria Indicator Surveys, both of which have the capacity to collect blood specimens, were completed under the DHS Program during this period (60). A total of 45 nationally representative Multi-Indicator Cluster Surveys were completed with UNICEF support during this same period (61). In other words, ∼21 nationally representative health surveys supported by either DHS or UNICEF were carried out each year. These and other health surveys could provide a platform for collecting information on MN statuses, so there are multiple opportunities for generating information on MN statuses in countries that could potentially benefit from this information. Adding MN status information to other survey platforms, like annual agricultural surveys, periodic household income and expenditure surveys, or dietary intake surveys could further expand potential data collection opportunities, although, with a few exceptions, these latter surveys do not currently collect clinical specimens.

### Reasons for the lack of information on population MN statuses

The International Zinc Nutrition Consultative Group and the Micronutrient Forum have collaborated to collect information on barriers and enablers for collecting information on MN biomarkers in the context of national nutrition and health surveys (62). A series of interviews and e-mail exchanges were completed with key informants involved in nationally representative surveys that included measurements of at least some MN status markers in Cambodia, Malawi, Pakistan, Uzbekistan, Ghana, and Uganda. In addition, interviews were conducted with representatives of several key agencies that support nutrition surveys (UNICEF, US CDC, ICF, and GroundWork). Based on responses to a preformulated list of questions, the authors of the report categorized factors that served as barriers or facilitators for including MN biomarker assessments in the surveys. The most important barrier cited by the country representatives was insufficient resources to cover the cost of the laboratory analyses. Other barriers that were mentioned less frequently were the lack of reliable, experienced, in-country laboratories; the inability to export clinical specimens due to government regulations; complexities in specimen collection and processing; tight time lines; and concerns that attention to MNs could undermine the data quality for other aspects of the survey. Several of the external experts also noted that a lack of awareness (both among the in-country decision makers and within the donor and technical support agencies) about the usefulness of MN status data for justifying and planning intervention programs is an important obstacle to leveraging the necessary funding. The main factors that favored inclusion of MN biomarkers in the surveys were the presence of supportive government authorities; an in-country “champion” (in a government, academic, or donor agency) to advocate for the information; the presence of a planned or ongoing MN deficiency control program for which baseline or follow-up information was desired; access to suitable laboratories; and availability of external experts to provide technical assistance to the local survey team.

Because more information is available for iodine biomarkers than for other MNs, it is instructive to explore the reasons for this relative degree of success. As noted above, iodine programs rely primarily on UIC data among school-age children (6–12 years of age) as an indicator of iodine exposure (63). Because most children in LMICs attend primary school, the specimens can be obtained at a common collection site, thereby facilitating specimen acquisition. Moreover, the ability to use urine rather than blood specimens reduces the level of invasiveness, thereby increasing survey participation, and simplifies the specimen collection and processing. Also, spectrophotometric analysis of UICs is fairly simple, and global quality assessment programs have been established (64). Finally, technical support is available from an experienced international community of clinicians (endocrinologists), nutritionists, and public health specialists through the IGN, and a data tracking system is publically available (52). These facts, coupled with the availability of a low-cost, widely implemented, and effective intervention (universal salt iodization), have made population assessments of iodine statuses relatively common. This experience, although not completely transferrable to other MN biomarkers, provides some insights into what will be required to achieve a similar degree of success.

### Increasing the availability of reliable data on MN statuses

**FIGURE 3:**
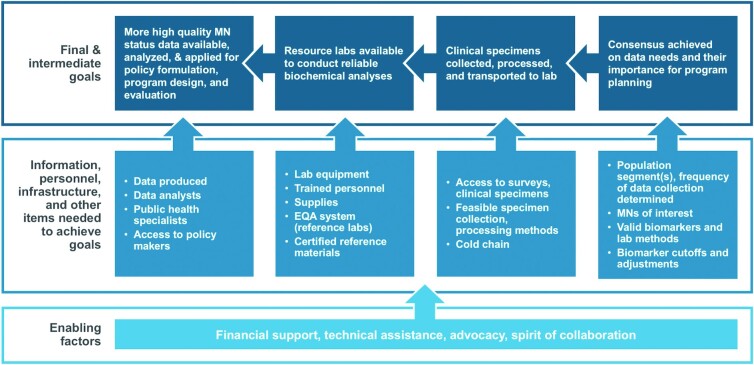
Theory of change for generating reliable data on population MN status globally. Abbreviations: EQA, External Quality Assessment; MN, micronutrient.

Considering the aforementioned obstacles and facilitators for obtaining the desired information, the working group developed a theory of change to indicate the steps needed to carry out this agenda, as well as the personnel, infrastructure, and other items required to achieve each of the intermediate goals (**Figure 3**). In many cases, there has been considerable progress in achieving the intermediate goals, but much remains to be accomplished to implement the full agenda.

The first step in this sequence of efforts is to: *1*) communicate with national policy makers and international donors about the importance of MNs for human health, the likely high prevalence of these deficiencies, and the current scarcity of relevant data; and *2*) discuss the need for greater availability and use of this information for MN deficiency control programs. This will require a deliberate and adequately resourced communication plan to deliver these messages and engage with key national decision makers and development partners. At the country level, available information from prior surveys, as well as suggestive information derived from national food balance sheets, dietary intake surveys, clinical case reports, and biomarker assessments and focused research studies using convenience samples, should be summarized to determine the MNs of possible concern in each setting and the population subgroups most likely to be affected. Appropriate biomarkers of the selected MNs and related laboratory methods should be described, along with an explanation of how this information can be applied to support program decision making. As part of this process, national champions and technical experts should be identified, as well as the need for any external technical and financial support to generate the information.

Once there is consensus on the need for MN status data and which types of information should be collected, the next step is to plan for obtaining the biological specimens in the context of a dedicated or shared survey platform. This requires collaboration and coordination with the survey team, planning the sampling design, working through the logistics of specimen collection and processing, and sensitization of the study population. After the samples are collected, they need to be transferred to a reliable laboratory for analysis. This, in turn, requires access to laboratories that are appropriately equipped and staffed to store samples, carry out the analyses, and participate in related external quality assessment schemes to guarantee the accuracy of the results. Finally, the resulting data must be analyzed, interpreted, and applied for policy formulation and program design and evaluation.

### Working group recommendations

Based on the background information and theory of change referenced above, the working group issued the following set of recommendations, as summarized in **Table 2**, leading with a statement that a formal, multi-component MN Data Generation Initiative should be launched to propel the full body of activities. The Initiative should be implemented at the global level by a management entity with relevant technical expertise, field experience, agility, and global reach and guided by appropriate technical experts and representatives of international agencies, nongovernmental institutions, and LMIC national governments. The management team should be responsible for advocacy and fundraising on behalf of the Initiative, as well as coordination among the partner agencies. The full working group report indicates possible entities that could implement specific components of the initiative (65).

**TABLE 2:** Summary of working group recommendations

Launch a multi-component, multi-stakeholder MN Data Generation Initiative
Establish a management team to lead and coordinate among stakeholders and implement selected tasks, including fundraising for the Initiative
Conduct advocacy, targeted both to in-country decision makers and program officers of development assistance agencies, to explain the importance of MNs, the need for MN status data, and methods for generating this information
Provide technical assistance to countries in survey design, specimen collection and laboratory analysis, and data analysis and interpretation
Create a central fund to help countries defray the costs of specimen collection and analysis
Develop consensus and disseminate information on preferred laboratory analysis methods for each MN biomarker
Establish regional resource laboratories to receive and analyze biological specimens collected during national nutrition surveys and to provide laboratory training
Expand and increase accessibility to external laboratory quality assessment programs and support preparation of certified reference materials where needed
Support a central data repository to archive survey results and periodically summarize these findings with regard to the global and regional prevalence of individual MN deficiencies, risk factors, and time trends
Track the progress of these activities of the Initiative and their impact on MN deficiency control programs

Abbreviation: MN, micronutrient.

In addition to the advocacy and communication activities noted above, a key recommendation is to establish a multi-donor, central fund and appropriate governance structure to assist countries with survey design and specimen collection, laboratory analyses, and data analysis and interpretation. The estimated budget for each component of the full range of activities is provided in the working group report. The group further recommends establishing 2 or more regional resource laboratories, initially in sub-Saharan Africa and South or Southeast Asia, that are able to receive and analyze biological specimens collected during national nutrition surveys. These resource laboratories should serve as both analytical and training facilities for the respective regions. The analyses should be standardized with respect to the specimen type (e.g., serum or plasma and type of anticoagulant) and analytical methods, and the laboratories should participate in external quality assessment schemes, which already exist for some MNs (64, 66, 67) but need to be established for others. In some cases, certified reference materials are lacking, so these should be made available for individual biomarkers. The initiative should provide support for a central data repository for curated survey results, building on the VMNIS and ultimately including appropriately anonymized, individual participant data. This information should be analyzed periodically to update regional and global estimates of the prevalences of MN deficiencies and risk factors for these deficiencies, with wide dissemination of the resulting information. Finally, a transparent tracking system should be established to monitor the progress of the initiative and its ultimate impact on MN deficiency control programs.

### Additional research needs

The proposed strategy for the MN Data Generation Initiative focuses primarily on advocacy and the provision of technical and financial support for MN status surveys, both to determine the need for public health programs and to permit more coherent program design and management. While developing this strategy, the working group identified several areas where additional research could help to advance this agenda. In particular, research is needed to identify novel biomarkers for some MNs and appropriate reference ranges and cutoffs to indicate both deficient and excess MN statuses and to confirm or refine recommended approaches for adjusting and interpreting these markers in the presence of inflammation. Second, efforts are needed to develop less invasive methods to collect biological specimens and minimize the amount of material that is required for laboratory analyses—for example, by using capillary blood collected with micro-sampling devices or as dried blood spots—and to reduce or eliminate the need for maintaining a cold chain. Although current technologies for analyses of the full array of recommended MN status biomarkers will continue to require the use of central resource laboratories for the foreseeable future, ultimately, point-of-collection analytical methods might be desirable to facilitate population assessment.

## Conclusions

Available information indicates that several key MN deficiencies are responsible for a sizeable share of the morbidity and mortality experienced by young children and women of reproductive age in LMICs. However, as confirmed by the current review, there is a scarcity of high quality, up-to-date information on population MN statuses; this lack of information undermines a full understanding of the magnitude of the problems and impedes the initiation, coherent design, and periodic reconceptualization of MN deficiency control programs. The working group identified key reasons for this lack of information and developed a set of recommendations to help close the data gap. Efforts are now underway to act on these recommendations and implement the proposed MN Data Generation Initiative; collective action and investments in generating more and better data on population MN statuses will be essential to plan and target intervention programs and monitor their progress.

## Supplementary Material

nqab173_Supplemental_File

## Data Availability

Data presented in the manuscript will not be made available because the findings described herein were derived from secondary analyses of data downloaded from the World Health Organization's Vitamin and Mineral Nutrition Information System (https://www.who.int/vmnis/database/en/) or provided by the International Zinc Nutrition Consultative Group. Thus, we defer to these organizations for information on data availability.

## Notes

This work was supported in part by the Bill & Melinda Gates Foundation (INV-003021). Under the grant conditions of the Foundation, a Creative Commons Attribution 4.0 Generic License has already been assigned to the Author Accepted Manuscript version that might arise from this submission. KSJ and SRM are supported by the National Institute for Health Research (NIHR) Cambridge Biomedical Research Centre (IS-BRC-1215-20014). The NIHR Cambridge Biomedical Research Centre is a partnership between Cambridge University Hospitals National Health Service (NHS)Foundation Trust and the University of Cambridge, funded by the NIHR. The views expressed are those of the authors and not necessarily reflect those of the NHS, the NIHR, or the Department of Health and Social Care.

Supplemental Table 1 is available from the “Supplementary data” link in the online posting of the article at https://academic.oup.com/ajcn/.

Abbreviations used: DHS, Demographic and Health Surveys; GNI, gross national income; IGN, Iodine Global Network; LMIC, low- and middle-income country; MN, micronutrient; NTD, neural tube defect; PSC, preschool children; UIC, urinary iodine concentration; VMNIS, WHO Vitamin and Mineral Nutrition Information System.
