# Supplementary material for: Increasing the availability and utilization of reliable data on population micronutrient (MN) status globally: the MN Data Generation Initiative
Source: Am J Clin Nutr. 2021 May 24;114(3):862–70. doi: 10.1093/ajcn/nqab173 (PMC8408880; doi:10.1093/ajcn/nqab173)
Supplement: nqab173_Supplemental_File [file nqab173_supplemental_file.docx]

**On-line Supplementary Material**

Increasing the availability and utilization of reliable data on population micronutrient (MN) status globally: the MN Data Generation Initiative

Brown KH, Moore SE, Hess SY, McDonald CM, Jones KS, Meadows SR, Manger M, Coates J, Alayon S, Osendarp SJM

**Supplementary Table 1:** Members of the Initiative’s Multi-Stakeholder Advisory Group and their institutional affiliations

| Seth Adu-Afarwuah | University of Ghana, Legon |
| --- | --- |
| Maria Andersson | Iodine Global Network; University Children’s Hospital, Zurich |
| Mandana Arabi | Nutrition International |
| Sufia Askari | Children’s Investment Fund Foundation |
| Shawn Baker | USAID |
| Gilles Bergeron | Nutrition Institute, New York Academy of Sciences |
| Eleanor Brindle | University of Washington |
| Kevin Cashman | University College, Cork |
| Nichola Connell | Eleanor Crook Foundation |
| Omar Dary | USAID |
| Jonathan Gorstein | Iodine Global Network |
| Ralph Green | University of California, Davis |
| Sarah Hanck | Rockefeller Foundation |
| Andreas Hasman | UNICEF |
| Rebecca Heidkamp | Johns Hopkins Bloomberg School of Public Health |
| Maria Elena Jefferds | US Centers for Disease Control and Prevention |
| Rolf Klemm | Helen Keller International; Johns Hopkins Bloomberg School of Public Health |
| Germana Leyrna | Tanzania Food and Nutrition Center |
| Cornelia Loechl | International Atomic Energy Agency |
| Renata Micha | Global Nutrition Report |
| Sorrel Namaste | The DHS Program, ICF |
| Lynnette Neufeld | Global Alliance for Improved Nutrition |
| Abigail Perry | UK Foreign, Commonwealth, and Development Office |
| Christine Pfeiffer | US Centers for Disease Control and Prevention |
| Felix Phiri | Malawi MOH |
| Rahul Rawat | Bill & Melinda Gates Foundation |
| Lisa Rogers | World Health Organization |
| Fabian Rohner | GroundWork Switzerland |
| Kyoko Shibata | World Bank |
| Ben Siddle | Irish Aid |
| Parminder Suchdev | Emory University |
| Masresha Tessama | Ethiopian Public Health Institute |
